# Anxious for the New Repertory

**Published:** 1885-11

**Authors:** S. Lilienthal


### ANXIOUS FOR THE NEW REPERTORY.

MY DEAR LEE:—My *Lippe's Repertory* is all torn, shall I get it rebound or can I soon hope to be the happy possessor of *two* copies of the new edition, one for my son and for me. A book so often used allows no partnership. Hope you enjoyed your trip. I just returned from Etruria and feel ready for work again. S. LILIENTHAL.

